# Long-Term Effects of Musical Training and Functional Plasticity in Salience System

**DOI:** 10.1155/2014/180138

**Published:** 2014-11-13

**Authors:** Cheng Luo, Shipeng Tu, Yueheng Peng, Shan Gao, Jianfu Li, Li Dong, Gujing Li, Yongxiu Lai, Hong Li, Dezhong Yao

**Affiliations:** ^1^Key Laboratory for NeuroInformation of Ministry of Education, School of Life Science and Technology, University of Electronic Science and Technology of China, Chengdu, China; ^2^Medical Engineering Department, PLA Chengdu Military Area Command General Hospital, Chengdu, China; ^3^Key Laboratory of Cognition and Personality of Ministry of Education, Southwest University, Chongqing, China

## Abstract

Musicians undergoing long-term musical training show improved emotional and cognitive function, which suggests the presence of neuroplasticity. The structural and functional impacts of the human brain have been observed in musicians. In this study, we used data-driven functional connectivity analysis to map local and distant functional connectivity in resting-state functional magnetic resonance imaging data from 28 professional musicians and 28 nonmusicians. Compared with nonmusicians, musicians exhibited significantly greater local functional connectivity density in 10 regions, including the bilateral dorsal anterior cingulate cortex, anterior insula, and anterior temporoparietal junction. A distant functional connectivity analysis demonstrated that most of these regions were included in salience system, which is associated with high-level cognitive control and fundamental attentional process. Additionally, musicians had significantly greater functional integration in this system, especially for connections to the left insula. Increased functional connectivity between the left insula and right temporoparietal junction may be a response to long-term musical training. Our findings indicate that the improvement of salience network is involved in musical training. The salience system may represent a new avenue for exploration regarding the underlying foundations of enhanced higher-level cognitive processes in musicians.

## 1. Introduction

The human brain is a complex and efficient information system. Learning changes this system as we adapt to information in the environment. Professional musicians comprise a special population for studying learning because they often begin intensive music training early in life and excel in various cognitive tasks, such as precise acoustic identification of musical sounds. Thus, musicians are an appropriate focus for neuroplasticity research about changes in functional and structural brain organization [1, 2]. In the 1990s, Schlaug and his colleagues showed neuroanatomical evidence of changes in brain structure associated with musical expertise and musicianship [3]. Since then, a growing number of structural imaging studies have reported increased volume of gray matter and altered diffusion parameters in the cerebellum, frontotemporoparietal cortex, corticospinal tract, and superior longitudinal fasciculus of musicians [2, 4]. Researchers have used a variety of functional neuroimaging tools to investigate neural plasticity in musicians. These studies support the idea of transfer effects, in which finely tuned gestural motor skills and heightened auditory perception, acquired through years of training, transfer to other domains, such as auditory processing, language, emotion, and attention [5–7]. Moreover, many studies have provided evidence for multidomain functional improvement in musicians, including enhanced motor skills, acoustic perception, emotion, and cognition [2, 8]. Thus, there may be a common foundation for the improvement of various cognitive functions in musicians.

The salience network comprised of the bilateral anterior insula, anterior temporoparietal junction (TPJ), and dorsal anterior cingulated cortex (ACC) has been observed in resting-state fMRI studies. The salience system is associated with detection of relevance among several interoceptive and exteroceptive stimuli and guides behavior while updating expectations about the internal and external environment [9, 10]. This system may play a role for fundamental cognitive and behavioral functions. As a result, it has become a topic of intensive research in recent years [10–12]. Of particular interest is coactivation of the insula and dorsal ACC, which has been observed during a variety of cognitive tasks. Menon and Uddin [12] recently proposed a model in which the salience network enables switching between the default mode network (DMN) and task-related brain networks. Once a salient stimulus is detected, the salience network facilitates task-related information processing by initiating appropriate transient control signals to engage brain areas mediating attention, working memory, and higher code cognitive processes and disengage DMN. Thus, we hypothesized that improved integration in the salience network would correlate with enhanced higher-level cognitive processes in musicians.

Recently, investigations of intrinsic functional connectivity based on resting-state functional magnetic resonance imaging (fMRI) have revealed several stable and reliable functional brain networks. Moreover, altered functional connectivity has been considered a potential biomarker for neuropsychiatric diseases, such as schizophrenia [13] and epilepsy [14]. Functional connectivity has also been used to describe plasticity induced by advanced level skill training, such as that required to achieve mastery of the game of chess [15], as well as long-term motor training [16, 17]. Our previous study found improved functional and effective connectivity of spontaneous intrinsic activity among multisensory and motor systems during the resting state in musicians [18]. These findings suggest that task-free analyses of intrinsic functional networks are useful for investigating the neural architecture relevant to musical training.

Information processing in the brain is dependent on interactions both between adjacent regions (local interactions) and between distant areas (distant interactions). Balance between the two types of interactions may contribute to the high efficiency of information processing in the brain [19]. Using local and global functional connectivity density (FCD) mapping known as a data-driven method, Tomasi and Volkow found that the strongest hubs in resting conditions were located in the DMN and sensory cortices [20]. Furthermore, strong local functional connectivity was observed in the primary sensory regions, motor regions, and the DMN, while preferential distant functional connectivity was observed in the DMN and heteromodal association area [19]. The nature of local cortical functional connectivity in musicians has been elusive. We hypothesized that local and distant functional connectivity would be enhanced by musical training, as this improvement might benefit performance while playing a music instrument.

To test this hypothesis, we evaluated the local and distant resting-state functional connectivity features by using a data-driven method. First, the FCD mapping was used to examine local functional connectivity associated with long-term musical training in musicians. Then, we assessed the distant functional connectivity of those regions in which we observed a significantly different local FCD. We predicted that regions associated with the salience system would show distinct local and distant functional connectivity. If confirmed, this pattern would support the role of the salience network in musical training and provide evidence for improved network integration as an underlying mechanism of enhanced higher-level cognitive processes in musicians.

## 2. Materials and Methods

### 2.1. Participants

Twenty-eight (20 female) musicians and 28 (20 female) nonmusicians participated in the study after providing informed written consent. These participants included two parts in which one was from the previous study [18] and the other was newly recruited in 2013. The experimental protocol was approved by the Research Ethics Review Board of Southwest University, China. All participants in the musician group were either music majors at Southwest University, China, or professional musicians who possessed an academic degree in music. Seventeen musicians had received long-term training in piano (6–20 years), and some of these individuals had also received training in either the Chinese zither or accordion. Eleven musicians primarily focused on violin (6–16 years). All nonmusicians were students at the University of Southwest China and reported that they had never received formal musical training or played any musical instrument. There was no significant difference in the years of education between the two groups (two-sample two-tailed *t*-test, *T* = 0.571, *P* = 0.182). All participants in both groups were right-handed and healthy, with normal brain structure, normal hearing, and no history of neurological or psychiatric deficits. All participants received monetary compensation for their involvement.

### 2.2. Image Acquisition

All participants were scanned in a 3T Siemens Trio Tim MRI scanner (Siemens, Erlangen, Germany) with an eight-channel phased array head coil. The experiments were conducted in the Key Laboratory of Cognition and Personality of Ministry of Education, Southwest University, China. The functional images were acquired using 2D gradient echo-planar imaging (EPI) sequences, with the following imaging parameters: thickness = 3 mm (with 1 mm gap), repetition time (TR) = 2,000 ms, echo time (TE) = 30 ms, field of view (FOV) = 22 × 22 cm, flip angle = 90°, and matrix = 64 × 64. A total of 205 volumes (32 slices per volume) were acquired during 410 seconds. To ensure steady-state longitudinal magnetization, the first five volumes were discarded. During data acquisition, participants were instructed to relax with their eyes closed, without falling asleep. Anatomical T1-weighted images were acquired using a three-dimensional- (3D-) spoiled gradient recalled sequence, generating 176 axial slices (thickness = 1 mm (no gap), TR = 8.5 ms, TE = 3.4 ms, FOV = 24 × 24 cm, flip angle = 12°, and matrix = 512 × 512).

### 2.3. Data Preprocessing Analysis

Preprocessing and analyses of fMRI data were carried out using SPM8 software (Statistical Parametric Mapping; http://www.fil.ion.ucl.ac.uk/spm). We conducted slice time correction, 3D motion detection and correction, and spatial normalization to the SPM EPI template and resampled the data (3 × 3 × 3 mm). Data were excluded if head motion exceeded 1 mm and 1° during fMRI acquisition. In addition, we also assessed group differences in translation and rotation of head motion using the following formula:

(1) $$\begin{array}{c} \frac{\text{head}\;\text{motion}}{\text{rotation}}=\frac{1}{M-1}\overset{M}{\underset{i=2}{\sum }}\sqrt{{\left|{\Delta d}_{x_{i}}\right|}^{2}+{\left|{\Delta d}_{y_{i}}\right|}^{2}+{\left|{\Delta d}_{z_{i}}\right|}^{2}}, \end{array}$$

where *M* is the length of the time series (*M* = 200 in this study), *x*_*i*_, *y*_*i*_, and *z*_*i*_ are translations/rotations at the *i*th time point in the *x*, *y*, and *z* directions, respectively, and Δ*d*_*x*_*i*__ = *x*_*i*_ − *x*_*i*−1_ and similarly for the other head motion/rotation parameters. There were no significant differences between the two groups in head motion and rotation (two-sample two-tailed *t*-test, *T* = 0.601, *P* = 0.275 for translational motion and *T* = 0.372, *P* = 0.644 for rotational motion). The averaged signals from white matter and cerebrospinal fluid, as well as the head motion parameters (three translations and three rotations), were corrected using a multilinear regression approach to minimize motion related fluctuations and other noise in the fMRI signals. We conducted temporal band-pass filtering (pass band 0.01–0.08 Hz) using a phase-insensitive filter, which served to reduce the effects of low-frequency drift and high-frequency physiological noise.

### 2.4. Local Functional Connectivity Analysis

We used degree centrality (or degree), which is a network measure, to map the local FCD for each voxel. In other words, the local FCD represented the number of voxels with significant connections in the local cluster around a given voxel. We used a key parameter Tc, the threshold of the correlation coefficient, to determine significant connections. Specifically, we conducted the following for a given target voxel. First, we calculated Pearson's linear correlation between the target voxel and its immediate neighbors. The voxels with significant links to the target were added to a cluster with the target voxel at the center. Next, we evaluated the relationship between the neighbors of the cluster and the target voxel, using the same threshold Tc. We used this process to extend the cluster until the correlation coefficient between the neighbors of the cluster and the target was less than Tc. Thus, the functional cluster had been established when the boundaries around the target voxel had been determined. All the voxels in the cluster were considered to have a significant connection with the target voxel. We then used the number of voxels (*K*) in the cluster surrounding the target voxel to map the local FCD for each target voxel.

We used the threshold Tc as a key parameter in our calculations. Although no gold standard exists in the previous literature, Tc = 0.6 is a common choice [20, 21]. Many brain network studies choose to use a dynamic threshold range, or multithreshold, because this produces more reliable and robust findings. We hypothesized that too small Tc would lead to increased type I error and too large Tc would lead to increased type II error. Thus, our thresholds ranged from 0.45 to 0.85 in 0.05 steps, such that nine thresholds were used in the current study. To address variability in the local functional connectivity strength of the voxels across participants, we normalized each individual local FCD map by 1/*k*_0_, where *k*_0_ represented the mean value across voxels in a given participant. We created nine normalized maps for the nine thresholds, Tc, for each participant. Finally, we performed spatial smoothing using a Gaussian kernel of full-width half-maximum (FWHM) 6 mm.

### 2.5. Group Analyses of Local Functional Connectivity

Group analyses of local FCD were conducted using *t*-tests. For each local FCD map for each threshold, Tc, we conducted a two-sample *t*-test comparing musicians and nonmusicians (*P* < 0.005). Correction for multiple comparisons was applied at the cluster level following Monte Carlo simulations conducted in the AlphaSim program [http://afni.nimh.nih.gov/pub/dist/doc/manual/AlphaSim.pdf]. To better detect differences between the two groups, we only included the clusters which were found significantly different between the two groups after five consecutive Tc values of FCD comparisons in the following analysis. Then, for each cluster in which a significant difference was found, the intersection was used as region of interest (ROI) in the subsequent functional connectivity analyses.

### 2.6. Functional Connectivity among ROIs

We adopted two strategies to evaluate the relationships among the regions with different local FCD between musicians and nonmusicians: functional connectivity analysis and global functional connectivity analysis.

First, we identified the time-courses of activation for the ROIs and calculated Pearson's linear correlation among these ROIs. After Fisher-z-transformation, one-sample *t*-tests were conducted within the musician and nonmusician groups. Then, we performed a univariate covariate analysis (ANCOVA) to detect the difference between the musician and nonmusician groups for all possible connections, and we adjusted the results for effects of age and gender.

Second, we conducted a functional connectivity analysis for the ROIs to investigate the functional connectivity in whole brain. The procedure was the same as in our previous studies [14]. In short, the regions that showed significant differences in local connectivity were selected as seeds. We calculated Pearson's correlation coefficients between the time-courses from each voxel and the averaged time-course of each seed and the Fisher-z-transformation for correlation coefficients.

As mentioned above, we conducted a one-sample *t*-test for each group and each seed. To compare the functional connectivity maps between the ROIs in the two groups, we calculated an *eta* ^2^ coefficient for each seed-map pair. The *eta* ^2^ represented the fraction of the variance in one signal accounted for by the variance in a second signal where the comparisons were done on a voxel by voxel basis [22]:

(2) $$\begin{array}{c} {eta}^{2}=1-\frac{\sum_{i=1}^{n}\left[{\left(a_{i}-m_{i}\right)}^{2}+{\left(b_{i}-m_{i}\right)}^{2}\right]}{\sum_{i=1}^{n}\left[{\left(a_{i}-\overset{-}{M}\right)}^{2}+{\left(b_{i}-\overset{-}{M}\right)}^{2}\right]}, \end{array}$$

where *a*_*i*_ and *b*_*i*_ represent the values at voxel *i* in functional connectivity maps *a* and *b*, respectively; *m*_*i*_ is the averaged value of the two images at voxel *i*, (*a*_*i*_ + *b*_*i*_)/2; *M*-bar is the grand mean value across all voxels in the mean image. Although the correlation coefficient *r* is often used for similarity descriptions of functional connectivity maps, we chose to use *eta* ^2^ because it focuses on the value of each voxel in the map and provides a better measure of the overall similarity or difference between two maps.

To compare the functional connectivity maps in terms of ROIs, we conducted a two-sample *t*-test on the maps which united the thresholded functional connectivity maps from the two groups for each ROI.

### 2.7. Correlation Analyses between Functional Connectivity and Music Training

To further explore the relationship between intrinsic functional connectivity and long-term music training, the further association analysis was performed. We used a partial correlation analysis to assess the relationship between extent of musical training and the functional connectivity features including the local FCD of each ROI and the functional connectivity between ROIs, controlling for the effects of age and gender.

## 3. Results

We excluded three of the musicians and two of the nonmusicians owing to excessive head motion. Thus, 25 musicians and 26 nonmusicians were included in the final analysis. The average age in the musician group was 23.13 years (SD = 2.38) and 21.93 years (SD = 2.05) in the nonmusician group. We found no significant difference (*P* = 0.15) in age between the two groups.

### 3.1. Local Functional Connectivity Analysis

**Figure 1:**
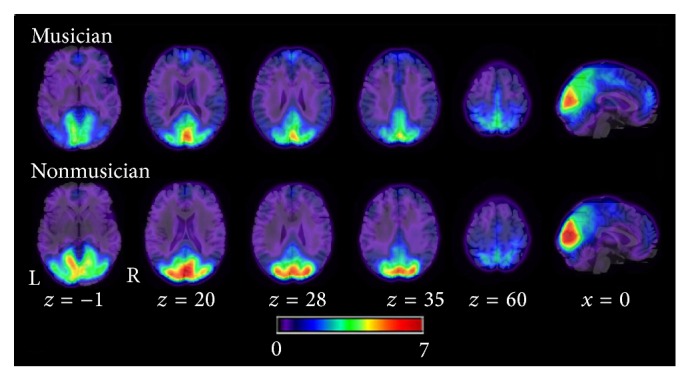
The local FCD maps in two groups (TC = 0.6). The upper part represents the averaged local FCD in musicians' group, and the bottom part represents averaged local FCD in nonmusicians' group. R, right; L, left.

**Figure 2:**
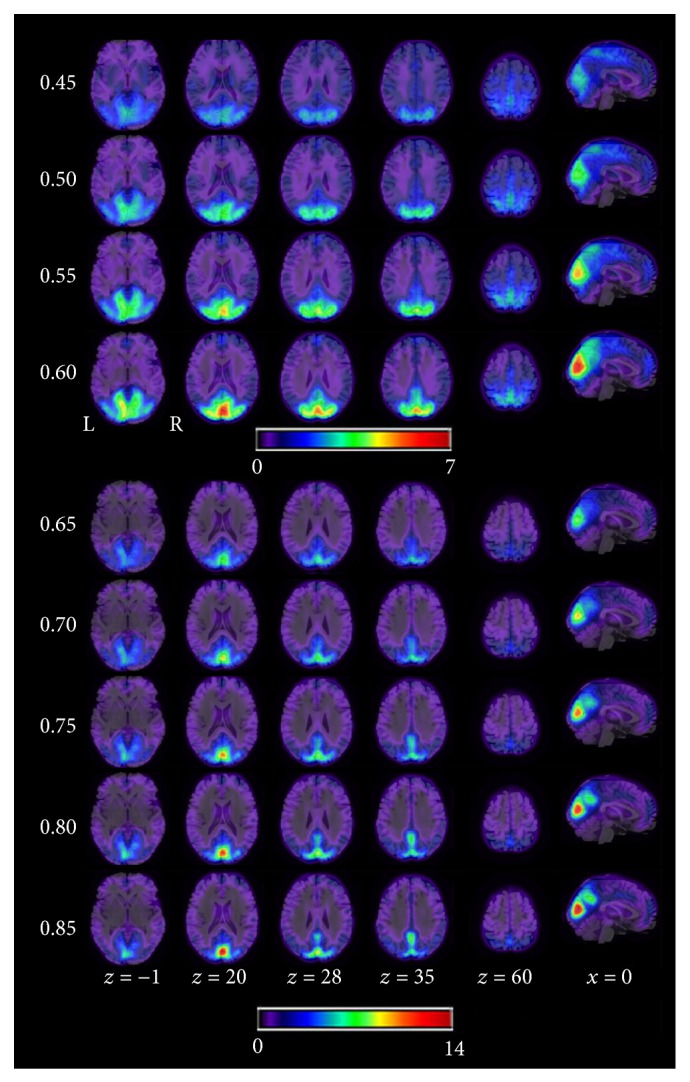
The mean local FCD maps across all subjects for 9 Tc thresholds.

Because “Tc = 0.6” is often chosen as a threshold when determining local FCD [20, 21], we first calculated the averaged distribution of the local FCD for “Tc = 0.6” in the two groups (musicians and nonmusicians; see Figure 1). We found the highest local FCD in the bilateral cuneus, precuneus, inferior occipital gyrus, cingulate cortex, precentral gyrus, middle temporal gyrus, middle frontal gyrus, inferior frontal gyrus, cerebellum, thalamus, and putamen. These findings were consistent with previous studies [20, 21].  Figure 2 shows the mean local FCD maps across all participants (musicians and nonmusicians) for every Tc threshold. For almost all Tc thresholds, we found the highest local FCD in the bilateral visual cortex, cuneus, and the medial prefrontal cortex. The patterns of local FCD were similar across Tc thresholds, although the size of the regions grew smaller as the Tc threshold increased.

**Figure 3:**
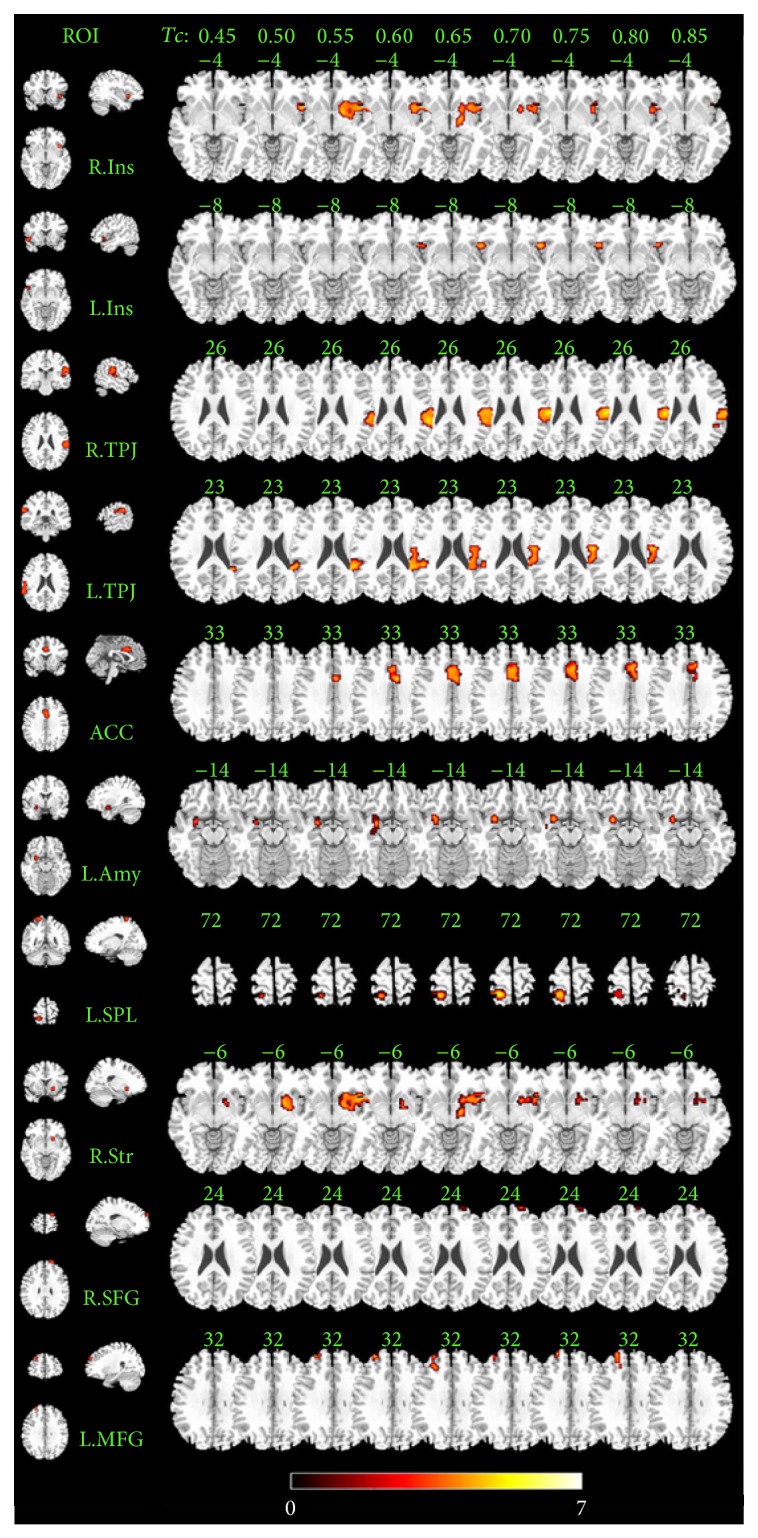
Significantly increased local FCD in musicians compared with nonmusicians. The left column shows 10 ROIs' position. The right part shows significantly increased local FCD in 10 axis images and 9 Tc values separately. ROIs' abbreviations are consistent with those shown in Table 1.

We conducted a two-sample *t*-test to compare data from musicians and nonmusicians for each threshold (*P* < 0.005, AlphaSim corrected). In order to get a stable difference between groups, we summarized all differences resulting from the comparison of FCD with nine Tc thresholds. We found that the local FCD had significantly increased for more than half of the thresholds (five of nine Tc values) at ten clusters in musicians compared with nonmusicians. These regions included the bilateral insula, bilateral TPJ, bilateral dorsal ACC, right striatum, right superior fontal gyrus, left middle frontal gyrus, left superior parietal lobule, and left amygdala (Figure 3). We also found a decreased local FCD in musicians at the occipital cortex in three Tc threshold conditions (Tc = 0.6, 0.65, and 0.7). In total, we identified 10 clusters for which there was a significantly increased local FCD in the musician group. These were identified as ROIs with distinct effects of musical training and included in the subsequent functional connectivity analysis. The center of these ROIs is shown in Table 1.

### 3.2. Functional Connectivity Analysis among ROIs

**Figure 4:**
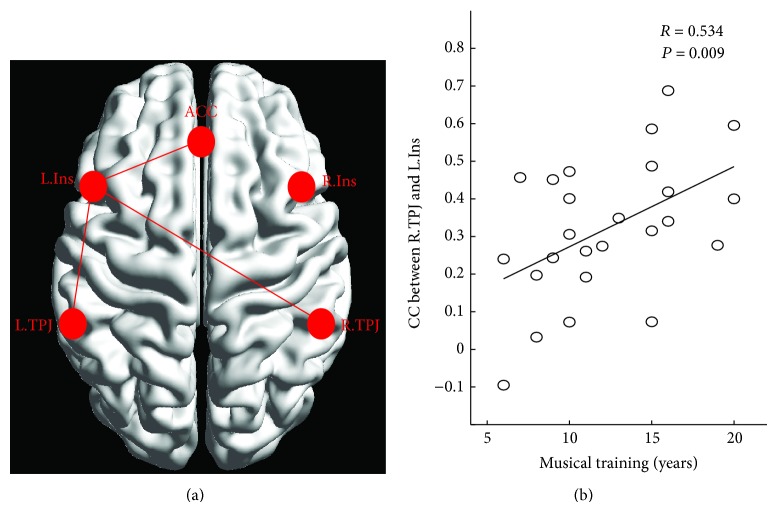
Significantly increased functional connectivity between ROIs in musicians compared with nonmusicians (a) and the relationship between the functional connectivity and musical training duration (b). ROIs' abbreviations are consistent with those shown in Table 1, and the abbreviation “CC” meant correlation coefficient.

We assessed the region-wise functional connectivity among ten ROIs in the two groups. We found that most of the ten ROIs were connected with each other in both participant groups (*P* < 0.05, FDR-corrected). We found full connections in the subnetwork comprising the dorsal ACC, insula, and TPJ, which have been identified as the salience network in previous resting-state fMRI studies [10, 12]. We then evaluated differences in this subnetwork between musicians and nonmusicians using a univariate ANCOVA. After controlling for the effects of age and gender, we found that three functional connections had significantly increased in musicians compared with nonmusicians (*P* < 0.05, FDR-corrected, Figure 4(a)), including one between the left insula and dorsal ACC (*F*(1,46) = 21.54), one between the left insula and left TPJ (*F*(1,46) = 7.91), and one between the left insula and right TPJ (*F*(1,46) = 8.86). We also tested the symmetry of the edge of the subnetwork constructed by 5 regions (Figure 4(a)). A paired *t*-test was conducted for three pairs of bilateral symmetry connections, including the connection between the TPJ and insula, the connection between the TPJ and ACC, and the connection between the insula and ACC. For example, for the connection between the TPJ and insula, the sample of matched pairs included the connection between left TPJ and left insula and the connection between right TPJ and right insula. We found significantly enhanced connection between right TPJ and right insula compared to that in left hemisphere in both groups (*P* < 0.0001 in both groups). It means the significant right-lateralized connectivity between the TPJ and insula. However, we found no difference in the lateralized index between the groups (*P* = 0.366). We found neither a lateralized predomination nor a difference between the musicians and nonmusicians in terms of connections between the TPJ and ACC or connections between the insula and ACC.

**Figure 5:**
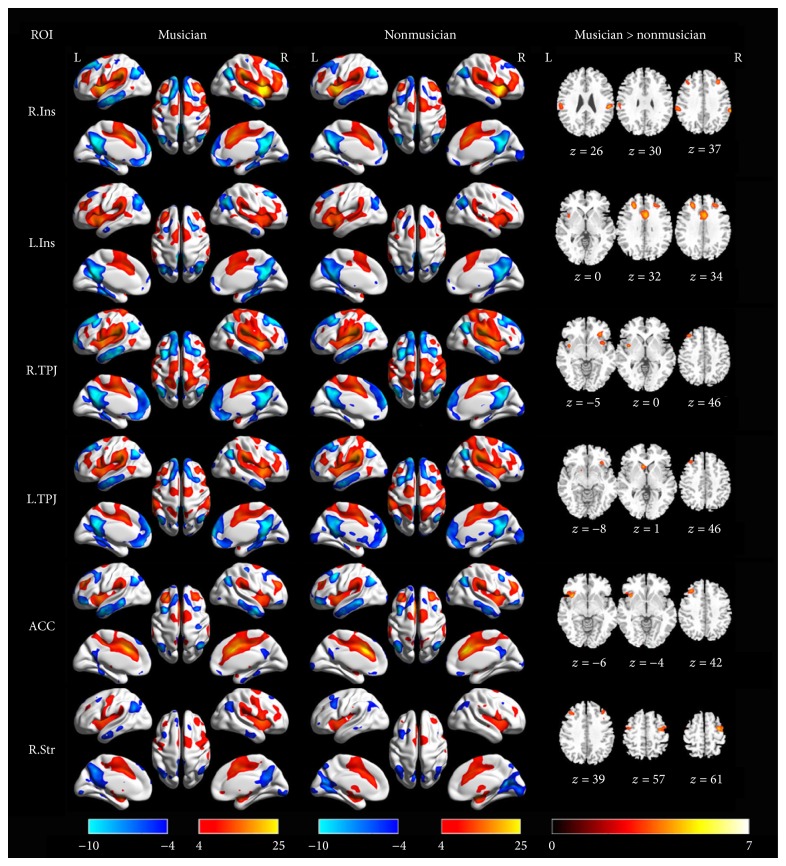
The group-level functional connectivity maps seeded at 6 ROIs and their difference between musicians and nonmusicians. The first column shows the seeds; the second and the third column illustrated the positive (hot color) and negative (cool color) functional connectivity with the seeds rendered onto a three-dimension brain reconstruction. The last column (axis images) represents significantly increased functional connectivity in musicians compared with nonmusicians. ROIs' abbreviations are consistent with those shown in Table 1.

**Figure 6:**
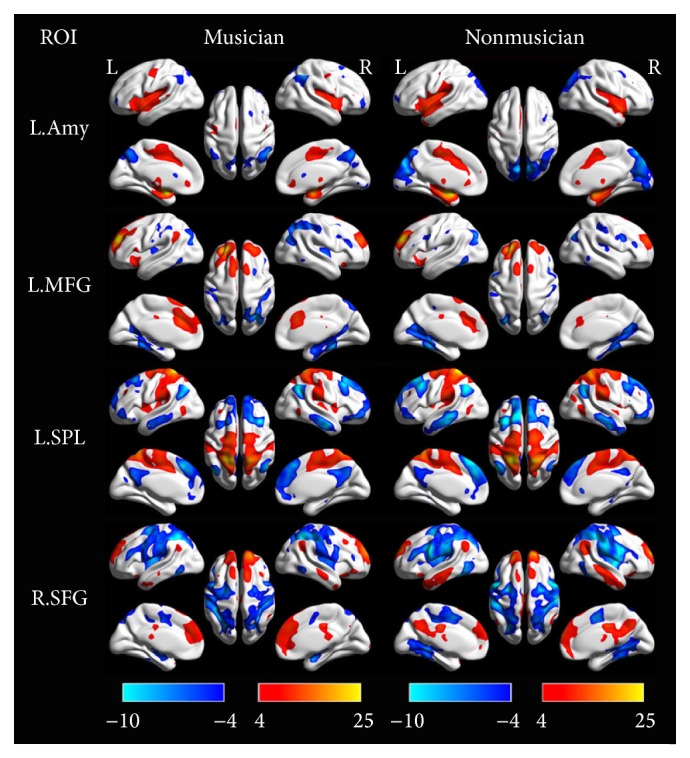
The group-level functional connectivity maps seeded at 4 ROIs. The first column shows the seeds; the second and the third column illustrated the positive (hot color) and negative (cool color) functional connectivity with the seed rendered onto a three-dimension brain reconstruction. ROIs' abbreviations are consistent with those shown in Table 1.

We generated a whole brain functional connectivity map for each group and each seed using a one-sample *t*-test in SPM8. These connectivity patterns were shown in Figures 5 and 6. Interestingly, these patterns were similar by visual inspection. The regions that showed a positive correlation with the seeds included the bilateral insula, TPJ, dorsal ACC, middle frontal gyrus, and supplementary motor area. In some cases, the bilateral thalamus and striatum were also included. The regions that showed a negative correlation with the seeds included the bilateral posterior cingulate cortex, medial prefrontal cortex, angular gyrus, and superior frontal gyrus, which were included in the DMN [23]. The *eta* ^2^ coefficient was used to assess the similarity of any two functional connectivity patterns at a voxel level. The result is shown in Table 2: we found a high similarity between connectivity patterns among all seeds, with the exception of two frontal seeds.

Compared with nonmusicians, musicians showed significantly enhanced functional connectivity in six ROIs (bilateral insula, bilateral TPJ, dorsal ACC, and right striatum, shown in Figure 5) and no significant differences in the other four ROIs (left amygdala, left middle frontal gyrus, left superior parietal lobule, and right superior fontal gyrus, shown in Figure 6).  Table 3 shows these findings in detail. In short, we observed increased functional connectivity among the bilateral TPJ, middle frontal gyrus, insula/frontal operculum, and ACC. We did not find decreased functional connectivity in any seeds. These findings from the seed-based functional connection analysis reflect increased connections among the major nodes of the salience network (ACC, TPJ, and insula).

### 3.3. Results of Correlation Analyses

For three connections with significant difference between groups, the correlation analyses showed that the functional connectivity between the left anterior insula and the right anterior TPJ was positively related to duration of musical training (*r* = 0.534, *P* = 0.009) when we controlled for the effects of age and gender (Figure 4(b)). For 10 ROIs, we did not find significant correlationship between duration of musical training and local FCD in all ROIs.

## 4. Discussion

We used resting-state fMRI to explore intrinsic functional connectivity in the brain of musicians. Combining local FCD, region-wise, and global functional connectivity analyses, we observed a distinct increase in the integration of the salience system in musicians. We found both a marked enhancement in local region functional connectivity and a significant increase in functional integration in the salience network of musicians. Components of the salience network seemed to be affected by musical training, especially the anterior insula, which has a critical and causal role in activating central executive networks and deactivating the DMN in response to salient stimuli [12, 24]. Considering these results, we propose that changes in the salience system trigger an improvement in higher-level cognitive processes in musicians. To the best of our knowledge, this is the first time that the salience system has been associated with musical training. More generally, our findings indicate that a data-driven approach to interpretation of resting-state functional connectivity data could be useful for evaluating cortical neuroplasticity related with musical training.

### 4.1. Local Functional Plasticity in Musicians

Several previous studies have reported structural alteration of brain tissue induced by musical training [25]. Specifically, increased volume of gray matter was found in motor, auditory, and visuospatial regions, which is reflective of different elements of musical experience, such as processing musical sounds and playing an instrument [4, 5]. Our FCD analysis revealed significantly enhanced local functional connectivity in the bilateral anterior TPJ, which is located at the ventral-anterior section of the inferior parietal lobule and surrounds the posterior end of the Sylvian fissure. The ventral intraparietal region is thought to contain many multimodal representations, including visual, auditory, and somatosensory information [26, 27]. As in our previous study [18], the current findings indicate that musicians possess increased integration in brain regions underlying motor and multiperceptional function. A previous study reported that musicians demonstrated greater activation in the anterior TPJ associated with auditory working memory compared with nonmusicians. Thus, the TPJ is implicated in auditory memory, which is crucial for learning music [28]. Our findings provide support for the notion that increased functional connectivity at the anterior TPJ is related with musical training.

The prefrontal cortex receives projections from both auditory and visual cortices and is known to play a role in various types of cognition, including temporal integration [29]. Although we observed an increase in local FCD, rather than in the connectivity between ROIs in the bilateral prefrontal cortex, we suggest that the local functional improvements at these regions may be relevant to multiperceptional function in musicians.

Emotions are a key element in our understanding of music. Previous fMRI studies have demonstrated that listening to music can affect the activity of many limbic and paralimbic structures [5, 30]. We found the increased FCD at amygdala, dorsal ACC, anterior insula, and ventral striatum. These regions are among the most commonly activated in functional neuroimaging experiments across both affective and cognitive domains [31, 32], such as in one study about music-evoked “chill” [7]. Here, we provide resting-state fMRI evidence illustrating improvements in functional connectivity in brain regions related to emotional processing in musicians. These brain areas may also play a consistent role in the emotional processing of music. A more recent meta-analysis showed leftward lateralization in the insula associated with affective processing [33]. The connectivity between the left insula and the amygdala has also been linked to anxiety levels in healthy controls [34]. We propose that the observed increase in local FCD at the left amygdala and the increase in functional connectivity related to the left insula are functionally coupled with respect to emotion processing related to the music. Therefore, our findings might contribute to understanding of the emotion modulation in music therapy.

### 4.2. The Salience Network: Potential Target of Musical Training

In the current study, we found not only enhanced local functional connectivity, but also increased distant functional connectivity among the regions that constitute the salience network. The salience network is considered to play important roles that are fundamental to cognition and behavior [10–12]. The often-observed coactivation of the insula and ACC across a variety of cognitive tasks suggests the existence of a functional network [35]. The amygdala is known to react to emotional and novel stimulation, suggesting a crucial role in salience processing [36]. In particular, connectivity between the anterior TPJ and the insula and cingulate cortex has been established. These regions are thought to comprise an externally oriented, stimulus-driven network that may modulate attention during salient events in our environment and guide our reactions [37–39]. In line with previous observations, our findings illustrate increased local functional connectivity between the components of the salience network.

The comparison between two groups revealed stronger connectivity between the left anterior insula and the bilateral anterior TPJ, ACC in musicians. Indeed, previous studies have reported rightward lateralization of the anterior insula and anterior TPJ in the salience- and attention-related networks [12, 38]. In line with previous findings [38], we found significant right-lateralized connectivity between the TPJ and insula in both groups. This finding suggests that right-lateralized ventral attention is strongly retained in musicians. Moreover, we found significantly increased connections between the left insula and left anterior TPJ in musicians. This is concordant with previous findings, which suggest that musical training leads to improved left anterior TPJ function, both in cross-sectional and in longitudinal design [40]. Therefore, the observed increase in functional connections to the left insula implicates the left insula, along with the right insula, in salience detection in musicians, which could lead to enhanced efficacy of the salience network. In addition, our observation of a positive correlation between the duration of music training and the connectivity between the left insula and right anterior TPJ further supports the hypothesis that musical training enhances functional integration of the left insula, which increases the efficiency of the salience network. Therefore, our findings reflect neural plasticity in musicians at a network level and implicate the salience network in musical training.

In general, the salience network works to identify important information from the vast and continuous incoming stream of sensory stimuli [12]. It partly overlaps with the right-lateralized ventral attention system, which is composed of the TPJ, ventral frontal cortex, and anterior insula [41]. This system shows increased activation upon detection of salient targets [42]. Once a stimulus is detected, the anterior insula facilitates task-related information processing by initiating appropriate transient control signals, which engage brain areas mediating attention, working memory, and higher order cognitive processes, while disengaging the DMN. The anterior TPJ has been proposed as the main component in this system. In addition, the right anterior insula enables switching between the default and task-related states of brain connectivity [24]. Considering the critical role of the anterior insula and anterior TPJ in high-level cognitive control and attentional processes, we suggest that the functional improvement in the salience network observed in musicians may contribute to the ability to rapidly relay bottom-up environmental information and intensify synergy of the salience network, enabling musicians to quickly detect relevant stimuli and produce appropriate behaviors.

There are two other possible interpretations regarding the role of the salience system in musical training. First, recent structural and functional network studies have revealed that the insula is rich club organization of human brain connectome [43]. The so-called rich club phenomenon in networks is said to be present when the highly connected (high-degree) hubs of a network are more densely connected among themselves than predicted on the basis of their high degree alone [44]. Attacks that specifically target richly connected brain areas might impair the global efficiency of a network more than those that affect random targets. Thus, we suggest that music training-induced changes in the salience system may be of low cost and highly efficient. The other possible reason concerns cross modal transfer effect plasticity. The transfer effects of years of musical training may result in enhanced processing in multiple domains that are not exclusively related to music [5]. Wan and Schlaug argued that the plasticity in regions of the parietal lobe in which multimodal integration takes place, such as the intraparietal sulcus, has an effect on related cognitive domains [8]. This is consistent with our finding of improved functional connectivity at the bilateral anterior TPJ, mainly at the intraparietal lobule. We propose to extend this view to apply to a network, instead of one region. Considering the above, we suggest that the salience system would be an optimal way for the human brain to respond to musical experience. The increased local and remote functional connectivity enabled by the salience network may contribute to the underlying mechanisms of enhanced higher-level cognitive processes in musicians.

### 4.3. Methodological Considerations

We applied a data-driven method to resting-state functional connectivity data to assess cortical neuroplasticity associated with musical training. Although local functional connectivity has been assessed in various studies [19–21, 45], the threshold of functional connection has yet to be concretely determined. Here, we used a set of successive thresholds, ranging from 0.45 to 0.85 in 0.05 steps, in the hope that this approach might yield more stable findings. We observed enhanced distant connectivity between the regions, with increased local connectivity in the musician group. Superficially, these findings are inconsistent in terms of system balance. However, similar preferential local and distant connectivity profiles have been reported in several cortical regions, such as the DMN [19, 45]. A potential interpretation of our findings is that salience information processing requires not only high local connectivity to sustain strong sensory constraints, but also a set of modular, tightly coupled areas to modulate efficient local processing, like that found in musicians. In other words, when salient information is detected, the processing system can simultaneously work on* in situ* information while associating distributed information with multiple regions. On the contrary, the combining two types of functional connectivity analysis for the same dataset may suffer from circular analysis [46]. In the future study, we will pay special attention to the underlying distortions.

One limitation of our study is that the number of participants was relatively small. The age and gender of the participants may have influenced our measure of functional connectivity, especially in terms of the local FCD [21]. A recent study of factors influencing maturational and musical training found age-related effects at the left TPJ, ventral premotor cortex, and intraparietal sulcus during music processing [47]. Our findings indicate increased functional connections with the left insula, including the connection between the left insula and left anterior TPJ, while controlling for the effects of age and gender. Future studies with a larger sample population are necessary to corroborate our findings and to detect the influence from gender. Another limitation is that changes in the salience network, which we have identified, may simply reflect altered coherence in the resting state and may not predict behavioral responses. This is certainly an issue for all resting-state studies and requires further investigation. Although there are some parallels between our findings and previous reports of stimulus-evoked changes in the regions of salience network in musicians [7, 40], multimodal designs may be useful in future investigations.

The individual variability related with the training, especially the variability in the level of expertise, should also be taken into account. The duration of musical training ranged from 6 to 20 years in this study. The large variability of expertise across subjects might lead to discrepant change of plasticity associated with training. The ongoing experience might aggravate the individual differences. However, it was difficult to group these subjects according to the duration of training. The correlation analysis strategy was adopted to identify the feature of plasticity within group. Moreover, the growing selection pressure promoting musicians to a more talented and conscientious stage would be another possible factor to encourage the individual variability. The interaction between the individual variability and training effects would be considered as a confounding factor in this study. For example, it has been found that the age of onset of training across the musicians affected the plasticity of brain [48]. The longitudinal further research would be included in the future.

## 5. Conclusion

In summary, we have demonstrated that data-driven methods applied to resting-state functional connectivity analyses can yield new data regarding cortical neuroplasticity in response to musical training. Our findings demonstrate enhanced functional connectivity in local regions and increased functional integration of the salience network in musicians. In addition, the observed increase in functional connectivity between the left insula and right anterior TPJ in musicians may be in response to long-term musical training. Our study provides the first evidence for the role of the salience system in musical training. We propose that improved integration in the salience system contributes to the underlying mechanisms of enhanced higher-level cognitive processes in musicians.

The further studies with multimodal and longitudinal designs are included in the future to yield the comprehensive understanding of brain related with musical experience. In addition, the alteration in salience system was also observed in neuropsychiatric disorders [49, 50]. Our findings, the improvement of salience system in musicians, may imply the role of the salience system in music therapy. The clinical research of music therapy should be included in the future to investigate our speculation.

## Figures and Tables

**Table 1:** Ten ROIs determined in local FCD analysis.

Regions	Abbreviation	Size of ROIs	Brodmann	MNI coordinate		
				*X*	*Y*	*Z*
Right insula	R.Ins	73	48	42	12	−9
Left insula	L.Ins	42	48	−43	16	−8
Right temporoparietal junction	R.TPJ	258	40,48	60	−22	23
Left temporoparietal junction	L.TPJ	161	40,48	−65	−33	28
Anterior cingulate cortex	ACC	113	24	6	0	30
Right striatum	R.Str	70		20	11	−9
Left amygdala	L.Amy	31		−27	6	−18
Left middle frontal gyrus	L.MFG	23	46	−29	53	32
Left superior parietal lobule	L.SPL	42	7,40	−21	−48	72
Right superior frontal gyrus	R.SFG	21	9,46	18	60	27

**Table 2:** The *eta* ^2^ coefficient between functional connectivity maps seeded at 10 ROIs in musicians and nonmusicians.

	R.Ins	L.Ins	R.TPJ	L.TPJ	ACC	R.Str	L.Amy	L.MFG	L.SPL	R.SFG
R.Ins		**0.851 **	**0.879 **	**0.910 **	**0.900 **	0.834	0.756	0.537	0.725	0.293
L.Ins	**0.895 **		**0.726 **	**0.775 **	**0.723 **	0.699	0.672	0.670	0.618	0.450
R.TPJ	**0.926 **	**0.854 **		**0.963 **	**0.849 **	0.758	0.704	0.379	0.875	0.158
L.TPJ	**0.941 **	**0.901 **	**0.957 **		**0.869 **	0.769	0.712	0.403	0.856	0.175
ACC	**0.859 **	**0.851 **	**0.822 **	**0.861 **		0.848	0.748	0.541	0.773	0.318
R.Str	0.862	0.820	0.839	0.844	0.823		0.885	0.556	0.662	0.371
L.Amy	0.743	0.754	0.763	0.746	0.742	0.920		0.455	0.558	0.364
L.MFG	0.529	0.670	0.424	0.480	0.640	0.506	0.461		0.373	0.820
L.SPL	0.720	0.685	0.856	0.819	0.721	0.684	0.641	0.341		0.208
R.SFG	0.319	0.414	0.220	0.240	0.398	0.322	0.319	0.830	0.179	

Notes: ROIs' abbreviations are consistent with those shown in Table 1. Upper-right part values result from the nonmusicians and bottom-left part values from musicians. The bold values cover five major nodes of salience network with high *eta* ^2^ value (*eta* ^2^ > 0.8).

**Table 3:** The significantly increased functional connectivity between musicians and nonmusicians in 6 seed-maps.

Seeds	Regions	MNI coordinates			Cluster	*T* value
		*X*	*Y*	*Z*		
R.Ins	R.Supramarginal	55	−25	26	40	4.86
	R.MFG	35	32	37	38	4.34
	L.Supramarginal	−60	−23	30	60	4.25
	L.MFG	−34	30	36	22	3.84

L.Ins	dACC	−1	12	32	208	6.54
	R.MFG	28	34	34	53	6.18
	L.MFG	−28	35	34	97	5.35
	L.Ins	−41	11	0	22	4.50

R.TPJ	L.Ins/frontal operculum	−40	3	0	66	5.79
	R.Ins	31	29	−5	31	5.35
	L.MFG	−24	33	44	26	4.79

L.TPJ	L.Caudate	−9	17	1	40	5.64
	R.Ins	32	25	−8	25	4.61
	L.MFG	−35	28	48	46	4.44

ACC	L.Ins/frontal operculum	−51	17	−6	74	5.39
	L.MFG	−34	30	42	78	5.05

R.Str	R.Precentral	41	−7	61	95	6.74
	L.Precentral	−47	−5	57	27	5.69
	R.MFG	37	32	39	39	5.58
	L.MFG	−36	30	39	40	4.45

Notes: ROIs' abbreviations are consistent with those shown in Table 1.
